# Letter from Warren Rowell

**Published:** 1850-10

**Authors:** Warren Rowell

**Affiliations:** 149 Madison-st., N. Y.


New York, *October* 11*th*, 1850.

*Dear Sir:*—In your last edition of "Principles and Practice of Dental Surgery," you have done me the honor to mention my case of supplying an artificial palate, and also copying the illustrations; but you have omitted my christian name. As there are several *Mr.* Rowells in New York, and as the Journal of Medicine may not be at hand for reference, I have to request you, if you have occasion to use my drawings, &c., in future, to do me the justice to add my christian name, and oblige

Yours, &c.

WARREN ROWELL.

To Chapin A. Harris, M. D. 149 *Madison-st., N. Y.*

Note.—We were not aware of the fact, until we received the above communication, that there were more than one dentist in New York by the name of Rowell; but should another edition of the work be called for, it will give us pleasure to comply with the request of Mr. R. In the mean time, we hope the publication of the above note will serve to correct any doubtful impression with regard to the real author of the operation, which the omission of the christian name may have made upon the minds of those into whose hands the work may have fallen.—*Editor.*

